# The gingival vein as a minimally traumatic site for multiple blood sampling in guinea pigs and hamsters

**DOI:** 10.1371/journal.pone.0177967

**Published:** 2017-05-22

**Authors:** Mariana Valotta Rodrigues, Simone Oliveira de Castro, Cynthia Zaccanini de Albuquerque, Vânia Gomes de Moura Mattaraia, Marcelo Larami Santoro

**Affiliations:** 1 Animal Facility, Instituto Butantan, São Paulo, São Paulo, Brazil; 2 Laboratory of Pathophysiology, Instituto Butantan, São Paulo, São Paulo, Brazil; International Nutrition Inc, UNITED STATES

## Abstract

Laboratory animals are still necessary in scientific investigation and vaccine testing, but while novel methodological approaches are not available for their replacement, the search for new, humane, easy, and painless methods is necessary to diminish their stress and pain. When multiple blood samples are to be collected from hamsters and guinea pigs, the number of available venipuncture sites–which are greatly diminished in these species in comparison with other rodents due to the absence of a long tail–, harasses animal caregivers and researchers. Thus, this study aimed to evaluate if gingival vein puncture could be used as an additional route to obtain multiple blood samples from anesthetized hamsters and guinea pigs in such a way that animal behavior, well-being or hematological parameters would not be altered. Thus, twelve anesthetized Syrian golden hamsters and English guinea pigs were randomly allocated in two groups: a control group, whose blood samples were not collected, and an experimental group in which blood samples (200 microliters) were collected by gingival vein puncture at weekly intervals over six weeks. Clinical assessment, body weight gain and complete blood cell count were evaluated weekly, and control and experimental animals were euthanized at week seven, when the mentolabial region was processed to histological analyses. Multiple blood sampling from the gingival vein evoked no clinical manifestations or alteration in the behavioral repertoire, nor a statistically significant difference in weight gain in both species. Guinea pigs showed no alteration in red blood cell, leukocyte or platelet parameters over time. Hamsters developed a characteristic pattern of age-related physiological changes, which were considered normal. Histological analyses showed no difference in morphological structures in the interdental gingiva, confirming that multiple blood sampling is barely traumatic. Thus, these results evidence that blood collection from multiple gingival vein puncture is minimally invasive and traumatic to hamsters and guinea pigs, and that it can be accomplished during at least six weeks.

## Introduction

The search for alternative methods and strategies for the reduction and improvement in the use of laboratory animals are practices that guarantee their care and ethical management [1–3]. In fact, the use of animals for educational and scientific purposes follow national and international regulatory guidelines, based on the "three R’s" principle: replacement, reduction and refinement [4].

Since blood collection is one of the most accomplished procedures in animal experimentation, choosing the appropriate venipuncture technique will depend on the species, the blood volume required for analysis, and the frequency of bleeding. As an approximation for rodents, the blood volume is 6–8% of body weight, and the maximum volume to be collected is approximately 10–15% of the total blood volume within the interval of 2 weeks, and 7.5% with the interval of 7 days [5,6]. However, these values have been experimentally reevaluated in mice, and higher percentages of total blood volumes can be safely taken from females, without adverse effects to them [7].

Guinea pigs (*Cavia porcellus*) and Syrian hamsters (*Mesocricetus auratus*) are extensively used as animal models for physiology, pathophysiology, periodontology, infectious diseases and vaccine testing [8–16]. In our institution, hamsters are used as models to study infectious diseases, whereas guinea pigs are regularly used to assess the immunogenic potency of diphtheria and tetanus vaccines, according to the recommendations of the Brazilian Pharmacopoeia [17].

Current guidelines for multiple blood collection in guinea pigs and hamsters are rather limited, once their anatomy is differentiated from other rodents, particularly by the presence of short tails and limbs. However, during the history of laboratory animal science, diverse venipuncture routes have been described for hamsters–the saphenous and cephalic veins [18–21], the left carotid artery [11], the cranial vena cava [22], the sublingual vein [23], and the retro-orbital sinus [24]–, and guinea pigs–the jugular vein [19], the cranial vena cava [19,25,26], the abdominal aorta [20], the caudal vena cava [20], the penile vein [20], the auricular vein [27,28], the retro-orbital sinus [29], the tarsal vein [30], the interdigital veins [31], and clipping of the toenails [32–34]. However, as the refinement of experimental conditions and the enhancement of animal care conditions have been progressively implemented, some techniques, such as clipping of toenails, are considered obsolete and inhumane nowadays. Moreover, by virtue of their short necks, blood sampling from the carotid artery, jugular vein, and cranial vena cava may unfavorably impact animal health or cause risk of mortality. Due to its inherent peril to animal welfare, cardiocentesis and abdominal aorta puncture cannot be used for multiple blood sampling, and they have been recommended only as a terminal procedure under general anesthesia. Blood sampling from the interdigital veins causes bruising and hematomas at the site of collection, adversely affecting animal welfare. In addition, due to relative small size of limbs in relation to the body size, particularly in guinea pigs, blood collection from saphenous and cephalic veins are invariably related to stressful restraining of animals. Blood collection from the retro-orbital sinus by untrained professionals, even under local and general anesthesia, may cause ocular injury, and is typically recommend only as terminal procedure [35]. Thus, the search for new, humane, easy and practical procedures for venous access is still required in these species.

The gingival vein (*labialis mandibularis* vein) has recently been described as an access route for i.v. inoculation and blood collection in anesthetized rats and mice. Around 800 μL of blood can be collected from rats and 100 μL from mice, with no apparent trauma to animals [36]. Considering that this route, for its ease of access, could also be used in guinea pigs and hamsters, we preliminarily compared the vascular structure of the mentolabial region in rats, guinea pigs and hamsters, and if the needle insertion and blood collection caused marked gingival lesion [37]. In pilot studies, we observed that the localization of this vein was similar in three species and that around 500 μL and 300 μL of blood could be collected, respectively, from guinea pigs and hamsters. The velocity of blood entering into the syringe allowed the accurate determination the volume of blood samples withdrawn. Moreover, since minor lesions were observed, those results suggested that this route could also be used for multiple bleedings.

Herein we investigated whether gingival vein puncture is a viable and humane alternative method to facilitate multiple blood collection in hamsters and guinea pigs. Animals were once weekly bled during six weeks, and monitored clinically, hematologically and for weight gain. At the end of blood samplings, histological analyses of the mandibular area were carried out. For blood collections, animals were anesthetized in order to minimize the stress of blood sampling and handling. The results evidenced that multiple blood sampling can be obtained using this route, without causing adverse effects to animals.

## Material and methods

### Animals and experimental procedures

Thirteen Syrian golden hamsters, thirteen English guinea pigs and one Wistar rat were from the Animal Facility, Instituto Butantan, São Paulo, Brazil, where the experiments were carried out. Animals were bred and maintained in a standardized environment, in rooms with defined flow of people, materials and supplies. In addition, they were continuously protected by health status barriers (barrier autoclave, HEPA air filtration system, differential pressure, etc). In addition, they were surveilled twice a year for the presence of pathogens, and the colonies were free of ectoparasites and the following microorganisms: (a) guinea pigs: *Bordetella bronchiseptica*, *Corynebacterium kutscheri*, β-hemolytic streptococci, *Streptococcus pneumoniae*, *Clostridium piliforme*, *Encephalitozoon cuniculi*, *Salmonella* spp, *Streptobacillus moniliformis*, *Chlamydophila caviae*, Pasteurellaceae bacteria, *Pseudomonas aeruginosa*, *Staphylococcus aureus*, *Yersinia pseudotuberculosis*, and dermatophytes; (b) hamsters: *Pasteurella pneumotropica*, *Clostridium piliforme*, *Corynebacterium kutscheri*, *Helicobacter* spp, and *Salmonella* spp. Room temperature was maintained at 22 ± 2°C, and humidity at 55 ± 10%. To control ammonia in the environment, the exhausting system was kept at 15 to 20 air changes per hour at room level. The light cycle was defined as 12-h light: 12-h dark. Animals were maintained in polypropylene cages–sizing 49 cm x 34 cm x 16 cm for hamsters, and 97 cm x 63 cm x 30 cm, without lids, for guinea pigs–, lined with autoclaved wood shavings. They had free access to drinking water and commercial pelleted feed: irradiated Nuvilab CR1 (Nuvital^®^, Quimtia, Brazil) for hamsters, and autoclaved Presence Cobaias^®^ (Presence, Brazil) for guinea pigs. The animal management routine comprised two weekly exchanges of cages, and fresh autoclaved water was changed three times per week. The facility is accredited by Conselho Nacional de Controle de Experimentação Animal (CONCEA). All procedures were in accordance with the National Guidelines [1] and were approved by the Institutional Animal Care and Use Committee of Instituto Butantan (CEUAIB 1122/ 13).

**Fig 1:**
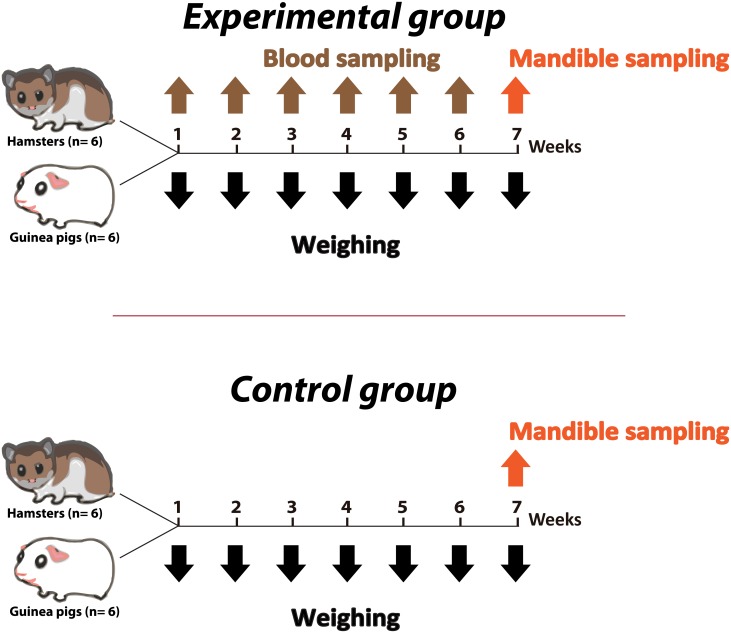
Schematic representation of timing of collection of blood samples, weighing and mandible sampling for histological analyses in control and experimental groups of hamsters and guinea pigs.

**Fig 2:**
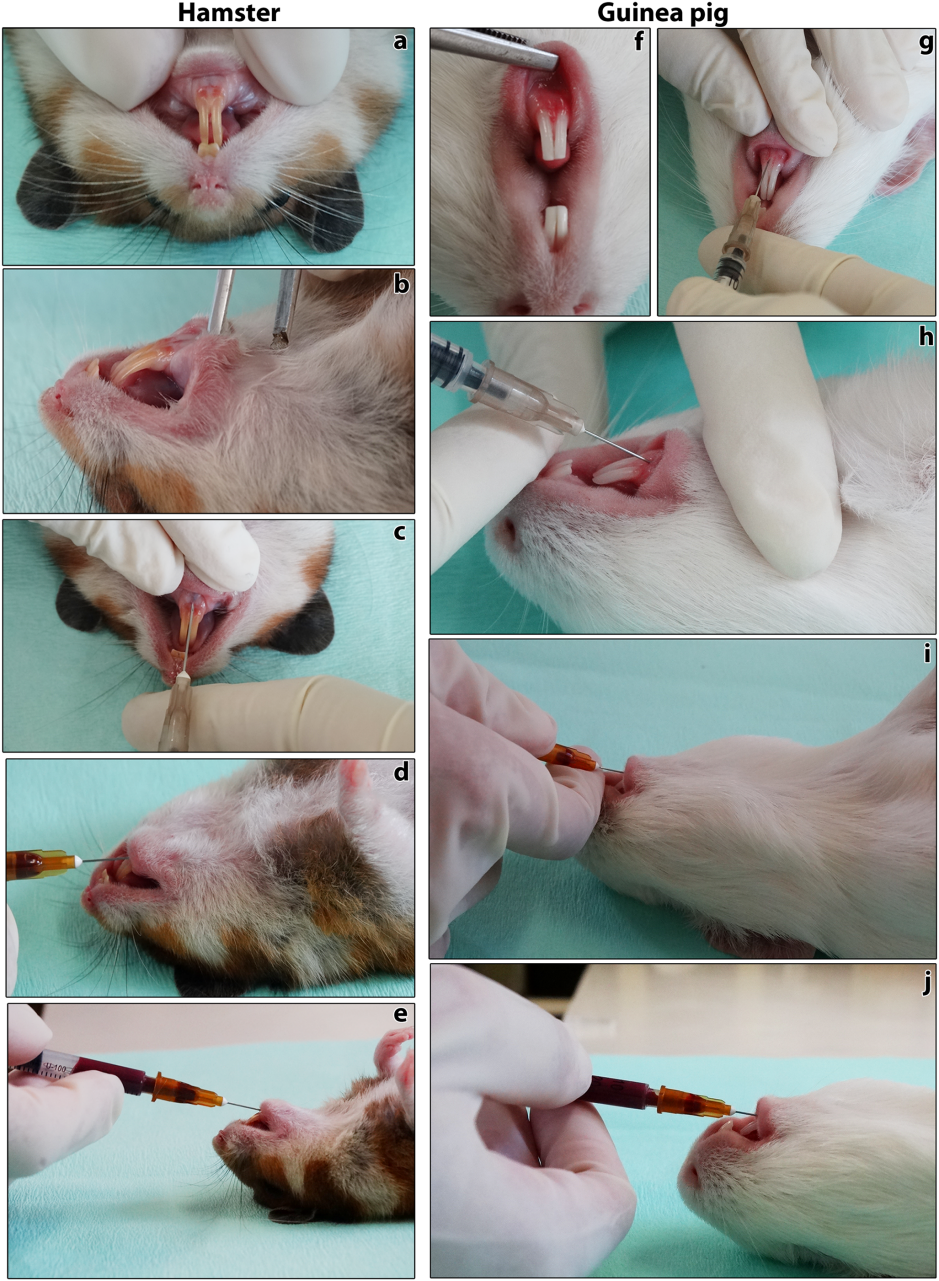
Blood collection from the gingival vein in hamsters (boxes a-e) and guinea pigs (boxes f-j). Notice the region for blood collection in hamsters (boxes **a** and **b**) and guinea pigs (box **f**). A 26G needle is inserted in both species (**c**, **g**, **h**). When blood comes in the needle hub, the left hand releases the lip (**d**, **i**), and gently pulls the syringe plunger to obtain blood samples (boxes **d**–**e**, and **i**–**j**). In single venipunctures, volumes higher than 400 μL can be obtained.

Twelve 4-week-old hamsters, six males and six females, with a mean initial weight of 72 g, and twelve 4-week-old guinea pigs, six males and six females, with a mean initial weight of 369 g were used. Animals were acclimated 72 h before the start of the experiment and were randomly divided into two groups for each species: the control group, composed of three males and three females, was anesthetized, but no blood sample was collected. The experimental group, also composed of three males and three females, was anesthetized, and six blood samples were collected from the gingival vein at weekly intervals, always at the same time (Fig 1). Animals were anesthetized i.p. with 10% ketamine (Vetnil^®^, Brazil) and 2% xylazine (Anesedan^®^, Brazil), with the following doses, respectively: hamsters 200 mg/kg and 10 mg/kg; guinea pigs 40 mg/kg and 5 mg/kg [38]. For blood collection, hamsters from the experimental group were maintained in dorsal decubitus on a table, and the inferior lip was pulled caudally. A 26G-needle attached to a 1.0 mL syringe was inserted 2–4 mm deep in the mucous region immediately below the gingival-incisor edge, towards the caudal direction, along the middle line between lower incisors, at an angle of 30° to 60° (Fig 2a–2e). For guinea pigs, the procedure was similar, except that the needle was inserted 3–5 mm deep at an angle of 35° to 60° (Fig 2f–2j). Aseptic procedures (use of disposable needles and syringes) were always employed during blood sampling.

Approximately 200 μL of blood was obtained over 2 min from both species, and blood was immediately transferred to 2-mL vials containing 2 μL of 10% Na_2_-EDTA. A complete blood cell count (CBC) was obtained in a BC-2800 Vet automatic cell counter (Mindray, China), in order to follow hematological alterations. After blood collection, sterile gauze was used to press the region where the needle was inserted for 1 min, and pressure was maintained until bleeding ceased. Thereafter, anesthetized animals were kept on a heating plate to control body temperature, and their behavior was closely observed until complete recovery. The behavior repertoire of each species was carefully observed, and animals were examined by a veterinarian. As an additional care, animals were weighed on digital scales at the same time at weekly intervals. As endpoints, we considered withdrawing animals from the experiment if either values of hemoglobin or hematocrit were below 20% of the baseline, or body weight loss was greater than 15%. However, these criteria were not fulfilled, and no animal was withdrawn during the experimental procedures. Blood was collected during six weeks, and in week seven all animals were euthanized by administrating an overdose of anesthetics (three times the anesthetic doses of ketamine/xylazine mixture for each species, as recommended by CONCEA [1]).

### Histological analyses

In order to evaluate whether gingival lesions occurred in the region of needle insertion, the mandible and the mentolabial region of all control and experimental animals were analyzed histologically. Briefly, animals were euthanized, and after the vital signs had ceased, the middle portion of the mandible, between the tip of the incisors and the temporomandibular joint, was cut with a pair of pliers. Thereafter, using surgical scissors, the tongue and other internal structures were separated from the cranium, and the rostral portion of the mandible was immediately immersed in Bouin's solution. In order to compare morphological structures, the same region was also obtained from one anesthetized rat and fixed and processed identically. The material was immersed in decalcifying solution (22.5% formic acid, 10% trisodium citrate, and 0.2% concentrated nitric acid) for around 6 days, and then processed for routine histopathological analysis. Sequential transverse 5-μm sections were prepared, with 25-μm intervals between each section, and the slides were stained with either hematoxylin-eosin (HE) or Carstairs’ method [39,40].

### Statistical analyses

The values of weight gain and CBC were submitted to analysis of homoscedasticity and normal distribution. Values of mean corpuscular hemoglobin (MCH), mean corpuscular hemoglobin concentration (MCHC), and mean platelet volume (MPV) were analyzed after log transformation. Two-way ANOVA with repeated measures was used to analyze differences in weight gain and hematological data. The software packages Stata^™^ 10.0 and SigmaPlot 12.0 were used for statistical analyses. Data were expressed as mean ± standard error of mean (s.e.m), and *p* values lower than 0.05 were considered statistically significant.

## Results and discussion

Taking into consideration that the three R’s are essential to humane animal research, herein we deliberately decided to use an anesthetic protocol that refined the procedure of blood collection in guinea pigs and hamsters, induced less stress, and alleviated the distress and pain after recovery from anesthesia. Although it has been reported that the ketamine/xylazine combination can adversely interfere in hematological parameters, at least in guinea pigs [41] and rats [42], this combination was our intentional choice for a general anesthesia protocol in guinea pigs and hamsters, once it is available worldwide, the prices are cheaper than isoflurane, it produces mild analgesia in hamsters and guinea pigs [43], and anesthetic machines are not required.

### Clinical and behavioral assessment of animals

During the experimental procedure, hamsters and guinea pigs remained well and did not manifest clinical signs of distress, aggressive or stereotyped behavior, lethargy, nor pain. After they recovered from anesthesia, they could stand, ambulate, eat and drink normally, and their posture and appetite were also normal. No evidence of sialorrhea, vomit, dehydration or paleness of mucous membranes was noticed. In addition, at the mentolabial region, we observed no signs of bruising, thrombosis, hemorrhage or scaring. Thus, we concluded, by the appearance and behavior of both species, that consecutive blood sampling through the gingival vein caused little hazard or distress to them.

**Fig 3:**
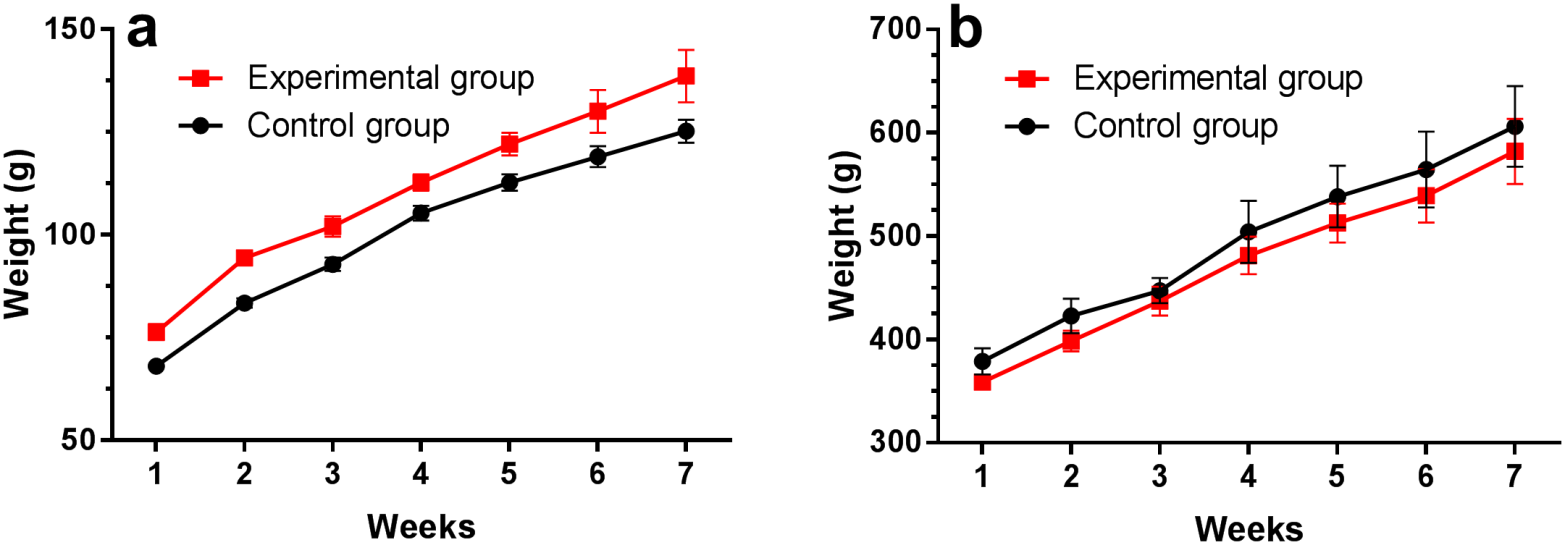
Body weight gain of hamsters (a) and guinea pigs (b) of experimental and control groups. Blood samples were taken from animals of the experimental group at weekly intervals, but not from the control groups. No statistically significant difference was noticed between groups over time. Data are expressed as mean ± s.e.m. (n = 6 per group).

In agreement with these observations, both species showed a similar increase in body weight during seven weeks, and no statistically significant difference has been noticed between mean weights of animals from the control and experimental groups (Fig 3a and 3b). Once body weight gain has been frequently used as an index of welfare in growing rodents [44,45], our results indicate that the procedure was neither traumatic to the oral cavity, which would prevent food ingestion, nor stressful to animals. Similar results were reported in rats and mice whose blood has been serially withdrawn [7,46]. The total blood volume obtained during six weeks from each animal was 1.2 mL, which corresponds to 1.5% and 0.3% of total blood volume of hamsters and guinea pigs, respectively, considering their mean initial weight. These percentages are below the maximum limit suggested elsewhere [5] for chronic blood sampling. In fact, studies of chronic bleeding of higher blood volumes than that undertook by us showed that the body weight gain was barely affected [7,46]. Moreover, potentially painful procedures, such as orchiectomy in guinea pigs, hardly disturb their body weight 48 h after surgery [45]. These data evidence that body weight gain does not remarkably change in animals that are under short-lived experimental procedures, so that it is not an appropriate methodology to evaluate distress in all contexts of animal experimentation.

### Hematological data

**Fig 4:**
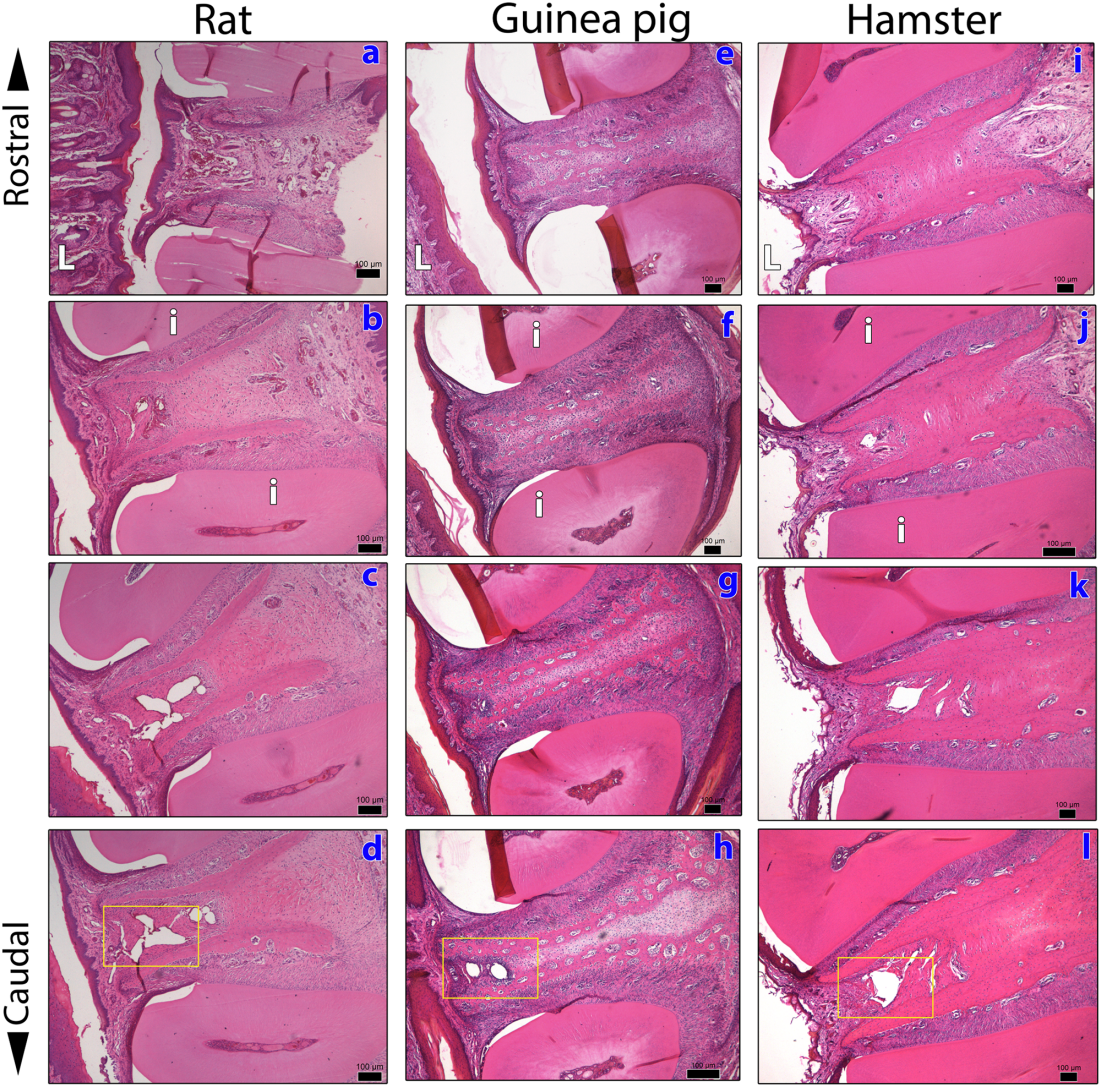
Photomicrographs of a series of transversal sections, in rostrocaudal direction, from the mentolabial and mandible region, depicting the gingiva between central incisors of one control rat (boxes a-d, in blue), one guinea pig (boxes e-h, in blue) and one hamster (boxes i-l, in blue). **i**- central incisors. **L**- inferior lip. The yellow outlines (boxes **d**, **h** and **l**) illustrate the region where the gingival vein is found. Note that the gingival vein is more rostral in rats than in hamsters and guinea pigs, and that it has a narrower lumen diameter in the rostral region in guinea pigs (box **h**). Sections were stained by HE. Bar sizes: 20 μm.

Tables 1 and 2 show the hematological data of hamsters and guinea pigs. It can be noticed that not all blood samples were represented in tables. In regard to hamsters, from 36 blood collections (Table 1), two samples were missed: one blood sample coagulated in the tube (week 1), and the other one was from an individual who died between week 5 and 6. During necropsy, no conspicuous abnormality was noticed, so that we could not identify the cause of death, but apparently it was not associated with the experimental protocol. With reference to guinea pigs, three blood samples coagulated (weeks 1, 3 and 6), and seven were in insufficient volume to automatic hematological counting. Compared with rats [36], hamsters and guinea pigs exhibited similar morphological structures at the mentolabial region, including those of the vascular network (Fig 4), but their gingival vein had narrower lumen diameters. In fact, unlike in rats, larger vascular lumina in guinea pigs and hamsters were found only in regions more profound and caudal (cf. Fig 4d, 4h and 4l). The largest lumen diameters of the gingival vein at the mentolabial region is approximately 120 μm in guinea pigs, 170 μm in hamsters and 200 μm in rats. These results confirm our practical experience that, contrasting with rats and hamsters, the needle has to be inserted 1–2 mm deeper in the interdental gingiva of guinea pigs. Thus, it is likely that the narrower lumen and the deeper localization of gingival vein make the blood collection more troublesome in guinea pigs, and may have caused obstacles to collect blood samples. This indicates that the professional has to gain confidence and a dexterous hand by training prior to be involved in a gingival venipuncture program in guinea pigs, and particularly, a cautious approach should be taken in regard to the angle of insertion of the needle in the gingiva.

**Table 1:** Follow-up of CBC values during once weekly blood collection from the gingival vein in hamsters.

Parameter*	Time					
	Week 1	Week 2	Week 3	Week 4	Week 5	Week 6
**RBC** (x10^12^/L)	6.43±0.20 (n = 5)	7.00±0.55(n = 6)	7.23±0.15 (n = 6)	7.75±0.26 (n = 6)	7.76±0.36 (n = 6)	8.93±0.21^§^ (n = 5)
**HGB** (g/dL)	13.6 ± 0.4 (n = 5)	14.4 ± 0.7 (n = 6)	14.7± 0.3 (n = 6)	15.5 ± 0.5 (n = 6)	14.9 ± 0.8 (n = 6)	16.4 ± 0.3 (n = 5)
**PCV** (%)	45.5 ± 1.5 (n = 5)	48.4±2.5 (n = 6)	47.5 ± 1.1 (n = 6)	49.4 ± 1.7 (n = 6)	48.8 ± 2.4 (n = 6)	54.8 ± 1.1 (n = 5)
**MCV** (fL)	70.8 ± 0.5 (n = 5)	70.3 ± 3.1 (n = 6)	65.8 ± 0.2^§^ (n = 6)	63.9 ± 0.3^§^ (n = 6)	62.9 ± 0.5^§^ (n = 6)	61.5 ± 0.5^§^ (n = 5)
**MCH** (pg)	21.1 ± 0.5 (n = 5)	20.9 ± 1.0 (n = 6)	20.3 ± 0.1 (n = 6)	19.9 ± 0.1 (n = 6)	19.1 ± 0.2^§^ (n = 6)	18.3 ± 0.2^§^ (n = 5)
**MCHC** (g/dL)	29.8 ± 0.9 (n = 5)	29.8 ± 0.3 (n = 6)	30.9 ± 0.1 (n = 6)	31.2 ± 0.0 (n = 6)	30.4 ± 0.2 (n = 6)	29.8 ± 0.1 (n = 5)
**RDW** (%)	13.2 ± 0.7 (n = 5)	12.0 ± 0.3 (n = 6)	11.9 ± 0.2 (n = 6)	11.7 ± 0.2 (n = 6)	9.9 ± 1.7^§^ (n = 6)	11.6 ± 0.1^§^ (n = 5)
**Platelets** (x10^9^/L)	418.2 ± 78.1 (n = 5)	471.8 ± 94.8 (n = 6)	792.8 ± 32.5^§^ (n = 6)	768.5 ± 46.3^§^ (n = 6)	638.3 ± 69.9 (n = 6)	739.6 ± 51.0^§^ (n = 5)
**MPV** (fL)	6.6 ± 0.4 (n = 5)	5.6 ± 0.2 (n = 6)	5.1 ± 0.1^§^ (n = 6)	5.1 ± 0.1^§^ (n = 6)	4.8 ± 0.1^§^ (n = 6)	4.8 ± 0.1^§^ (n = 5)
**PDW** (%)	19.1 ± 0.4 (n = 5)	18.0 ± 0.4 (n = 6)	17.4 ± 0.1^§^ (n = 6)	17.3 ± 0.2^§^ (n = 6)	17.3 ± 0.2^§^ (n = 6)	16.8 ± 0.2^§^ (n = 5)
**WBC** (x10^9^/L)	7.44±0.74 (n = 5)	6.50±1.04 (n = 6)	7.22±0.73 (n = 6)	7.03±1.11 (n = 6)	4.52±0.48 (n = 6)	7.26±1.55 (n = 5)

*Abbreviations: **RBC**: red blood cell count; **HGB**: hemoglobin concentration; **PCV**: packed cell volume; **MCV**: mean corpuscular volume; **MCH**: mean corpuscular hemoglobin; **MCHC**: mean corpuscular hemoglobin concentration; **RDW**: red cell distribution width; **MPV**: mean platelet volume; **PDW**: platelet distribution width; **WBC**: white blood cell count.

^§^Statistically significant different (*p*< 0.05) in comparison with the baseline value (Week 1). Data are expressed as mean ± s.e.m.

**Table 2:** Follow-up of CBC values during once weekly blood collection from the gingival vein in guinea pigs.

Parameter*	Time					
	Week 1	Week 2	Week 3	Week 4	Week 5	Week 6
**RBC** (x10^12^/L)	5.54 ± 0.49 (n = 4)	5.11 ± 0.11 (n = 5)	6.01 ± 0.59 (n = 3)	5.06 ± 0.22 (n = 5)	4.91 ± 0.21 (n = 5)	5.22 ± 0.49 (n = 4)
**HGB** (g/dL)	13.9 ± 1.3 (n = 4)	13.0 ± 0.3 (n = 5)	15.4 ± 1.4 (n = 3)	12.6 ± 0.6 (n = 5)	12.1 ± 0.4 (n = 5)	12.5 ± 1.6 (n = 4)
**PCV** (%)	45.6 ± 4.2 (n = 4)	42.4 ± 1.0 (n = 5)	49.9 ± 4.1 (n = 3)	42.4 ± 1.5 (n = 5)	41.0 ± 1.3 (n = 5)	43.0 ± 4.2 (n = 4)
**MCV** (fL)	83.8 ± 1.7 (n = 4)	83.2 ± 0.9 (n = 5)	83.4 ± 1.7 (n = 3)	84.1 ± 1.4 (n = 5)	83.8 ± 1.1 (n = 5)	82.5 ± 0.9 (n = 4)
**MCH** (pg)	25.5 ± 0.4 (n = 4)	25.4 ± 0.4 (n = 5)	25.7 ± 0.7 (n = 3)	24.8 ± 0.3 (n = 5)	24.7 ± 0.2 (n = 5)	23.7 ± 1.1 (n = 4)
**MCHC** (g/dL)	30.5 ± 0.2 (n = 4)	30.6 ± 0.2 (n = 5)	30.9 ± 0.3 (n = 3)	29.6 ± 0.3 (n = 5)	29.5 ± 0.3 (n = 5)	28.7 ± 1.1 (n = 4)
**RDW** (%)	15.5 ± 0.3 (n = 4)	13.5 ± 0.6 (n = 6)	12.2 ± 0.7 (n = 3)	14.1 ± 0.4 (n = 5)	14.0 ± 0.8 (n = 5)	13.4 ± 0.9 (n = 4)
**Platelets** (x10^9^/L)	397.8±34.6 (n = 4)	410.8± 38.4 (n = 5)	276.7± 39.0 (n = 3)	316.0± 49.9 (n = 5)	341.8± 62.3 (n = 5)	358.5± 70.2 (n = 4)
**MPV** (fL)	4.9 ± 0.2 (n = 4)	5.6 ± 0.3^§^ (n = 5)	5.3 ± 0.1 (n = 3)	4.7 ± 0.1 (n = 5)	4.5 ± 0.1 (n = 5)	4.6 ± 0.2 (n = 4)
**PDW** (%)	16.3 ± 0.2 (n = 4)	16.3 ± 0.2 (n = 5)	15.9 ± 0.1 (n = 3)	15.8 ± 0.1 (n = 5)	16.0 ± 0.1 (n = 5)	16.2 ± 0.2 (n = 4)
**WBC** (x10^9^/L)	9.85±1.91 (n = 4)	10.82±1.15 (n = 5)	8.34±0.43 (n = 3)	8.00 ± 0.56 (n = 5)	7.96±1.06 (n = 5)	7.40 ± 1.31 (n = 4)

*Abbreviations: **RBC**: red blood cell count; **HGB**: hemoglobin concentration; **PCV**: packed cell volume; **MCV**: mean corpuscular volume; **MCH**: mean corpuscular hemoglobin; **MCHC**: mean corpuscular hemoglobin concentration; **RDW**: red cell distribution width; **MPV**: mean platelet volume; **PDW**: platelet distribution width; **WBC**: white blood cell count.

^§^Statistically significant different (*p*< 0.05) in comparison with the baseline value (Week 1). Data are expressed as mean ± s.e.m.

No animal showed conspicuous leukopenia, leukocytosis or anemia, as well as no animal was withdrawn from this study based on the endpoint criteria. Moreover, statistically significant differences between males and females were not noticed for erythron, leukocyte and platelet parameters at any time interval in both hamsters and guinea pigs.Thus, the changes described below were of minor intensity.

In regard to white blood cell counts (WBC), they were within the normal reference ranges [47], showing that no infection or severe inflammation resulted from successive blood collection.

Anemia was not detected in the experimental groups over time, once values for red blood cell counts (RBC), hemoglobin concentration and packed cell volume (Tables 1 and 2) were within the normal reference ranges for hamsters and guinea pigs [47]. In hamsters, nonetheless, RBC values did show a statistically significant increase (*p*< 0.01) at week 6 compared to week 1 (Table 1), whereas a mild progressive drop (*p*< 0.05) was noticed in values of MCV from week 3, of MCH from week 5, and of red cell distribution width (RDW) from week 5. Such trends suggested that mild iron deficiency was occurring, but since blood volumes withdrawn from animals were so modest, this reasoning could not explicate such changes. However, checking normal reference values for hamsters, we observed that their hematological data do change physiologically with age [48–51], so that from 4 weeks old to about 8–9 week old, the time interval in which we studied them, there is an increase around 50% in RBC count [50], ca. 10% in hematocrit [49,50], and ca. 10% in hemoglobin levels [49,50]. Moreover, there was a decrease ca. 20% in MCV and ca. 15% in MCH [50]. All these findings are in agreement with our results, evidencing that the changes observed in hamsters are physiological. Likewise, guinea pigs did not show marked difference in RBC parameters either (Table 2). Thus, multiple blood sampling over six weeks induces no important change in the erythrogram of hamsters and guinea pigs. In fact, the removal of higher percentages (15–25%) of total blood volume in other laboratory animals evoked no iron deficiency anemia, and the recovery of erythron values was complete few days after the conclusion of blood sampling [7,46,52].

Platelet counts and mean platelet volume (MPV) values also raised significantly from week 3 (*p*< 0.05) compared to baseline values and the normal reference range for hamsters [47], and, simultaneously, platelet distribution width (PDW) values decreased (*p*< 0.05) in regard to baseline values. As far as we know, no report has dealt with such changes in platelet parameters during hamster growth. On the other hand, guinea pigs exhibited no relevant change in regard to platelet parameters over time.

### Histological analyses

Histopathological analyses of the gingiva between inferior incisors (interdental gingiva) from guinea pigs and hamsters predominantly showed normal structures both in control and experimental groups. Generally, it was noticed absence of marked inflammatory reaction, degenerative lesions, necrotic areas or any other features implying tissue injury due to needle insertion.

**Fig 5:**
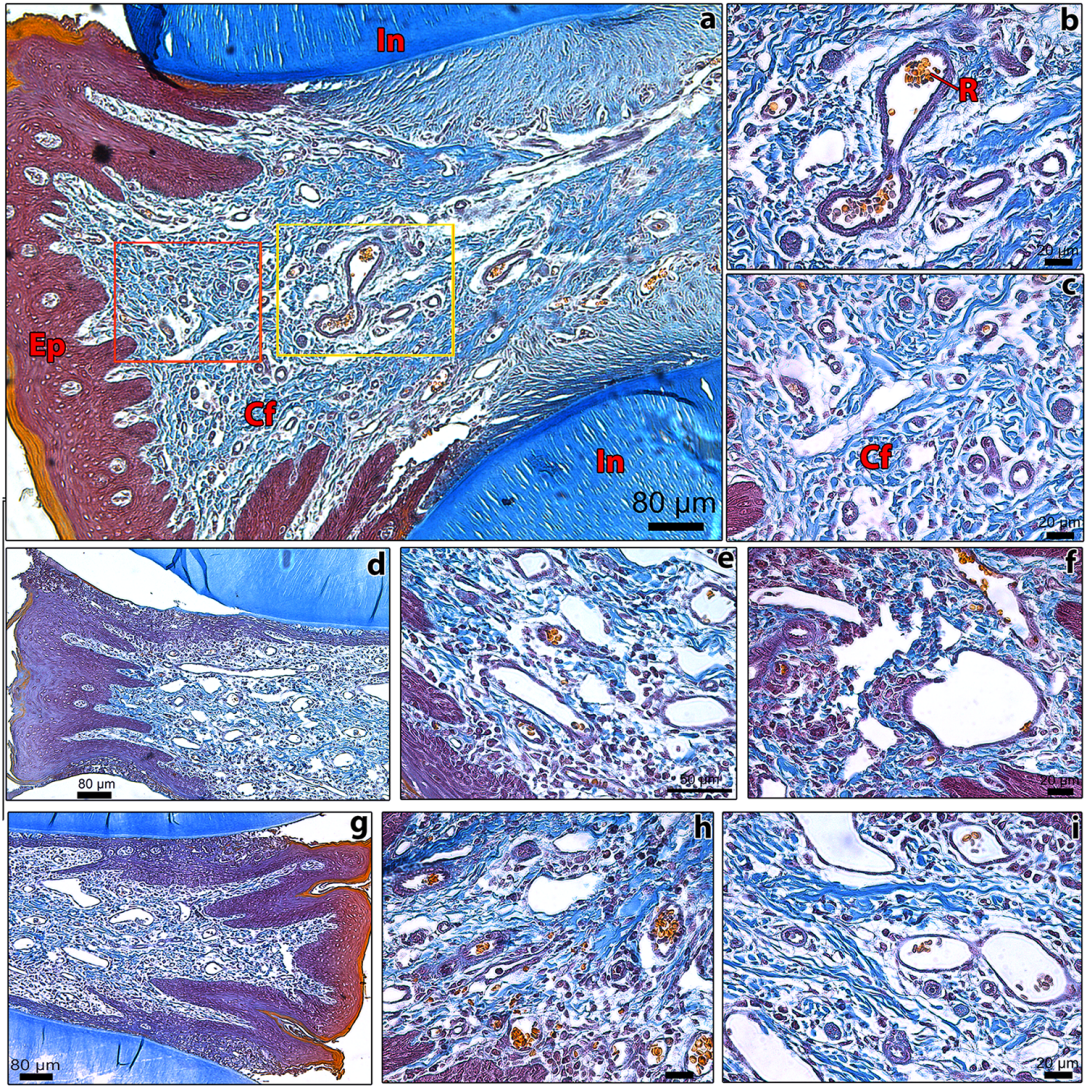
Photomicrographs of transversal sections from the mentolabial region of guinea pigs from experimental (boxes a-e, g-i) and control (box f) groups, depicting the gingiva between central incisors (In). The needle was inserted in the rostral region of the interdental gingiva of experimental animals (boxes **a**–**e**, **g**–**i**); control guinea pigs (box **f**) were not phlebotomized. Sections were stained by Carstairs’ method: collagen fibers (**Cf**) are stained in celeste blue, epithelium (**Ep**) in magenta/purple, red blood cells (**R**), and keratin in yellow. The area around the gingival vein delineated by the yellow frame in box **a** is magnified in box **b**. Box **c** is the magnification from the region enclosed by the red box delineated in box **a**. In boxes **b** and **c**, notice the normal distribution and quantity of collagen fibers, and the absence of polymorphonuclears, mononuclear cells, and inflammatory exudate in the lamina propria; red blood cells were also evidenced inside blood vessel lumina, in both control (box **f**) and experimental animals. Boxes **d** and **e** show the same rostral area from different experimental animals. No morphological differences were noticed in regard to control animals (box **f**). Boxes **g**–**i** depict the more caudal region in the interdigital gingiva, close to the tongue, where the needle did not reach; notice that the same pattern of distribution of collagen fibers in lamina propria and epithelium integrity in both caudal and rostral regions, evidencing that wound healing was effective and rapid in experimental animals. Boxes **a**, **d**, **g**, bar size = 80 μm; boxes **b**, **c**, **f**, **h**, **i**, bar size = 20 μm; and box **e**, bar size = 50 μm.

The gingiva between the inferior central incisors in control and experimental guinea pigs showed that it was lined by thick squamous epithelium in rostral (close to the inferior lip) and caudal (close to the tongue) regions, and that the epithelium circumscribing the incisors was thinner (Fig 5). Under the epithelium, the lamina propria was comprised of connective tissue rich in collagen fibers, blood vessels–frequently filled with RBC–, and nerve fibers. In transversal serial sections from more caudal regions, bone tissue (mandibular bones) was also noticed inside the lamina propria. In more rostral sections, where the needle was inserted, there was a typical pattern of distribution of cells and collagen fibers. Large numbers of small blood vessels and nerve fibers were observed along the serial sections, evidencing the abundance of blood and nerve supply to gingiva, as expected (Fig 5). One week after the last blood collection in the experimental group, few polymorphonuclears or mononuclear cells were observed in the lamina propria. Likewise, no important differences were detected in squamous epithelium and lamina propria in animals from the experimental group, showing no signs of laceration or poor wound healing. In addition, it was observed normal cell numbers, collagen distribution pattern, and quantity of blood vessels in lamina propria in comparison to controls. However, the presence of inflammatory cells adhered to vessel walls was noticed in only one animal from the experimental group; in fact, this guinea pig was the only one to show macroscopic edema in the region of blood collection in *post-mortem* examination. Altogether, these results indicate that minute histological alterations were observed in gingival structures of animals from the experimental group, implying that irrelevant morphological alterations were induced by once weekly venipuncture in guinea pigs.

**Fig 6:**
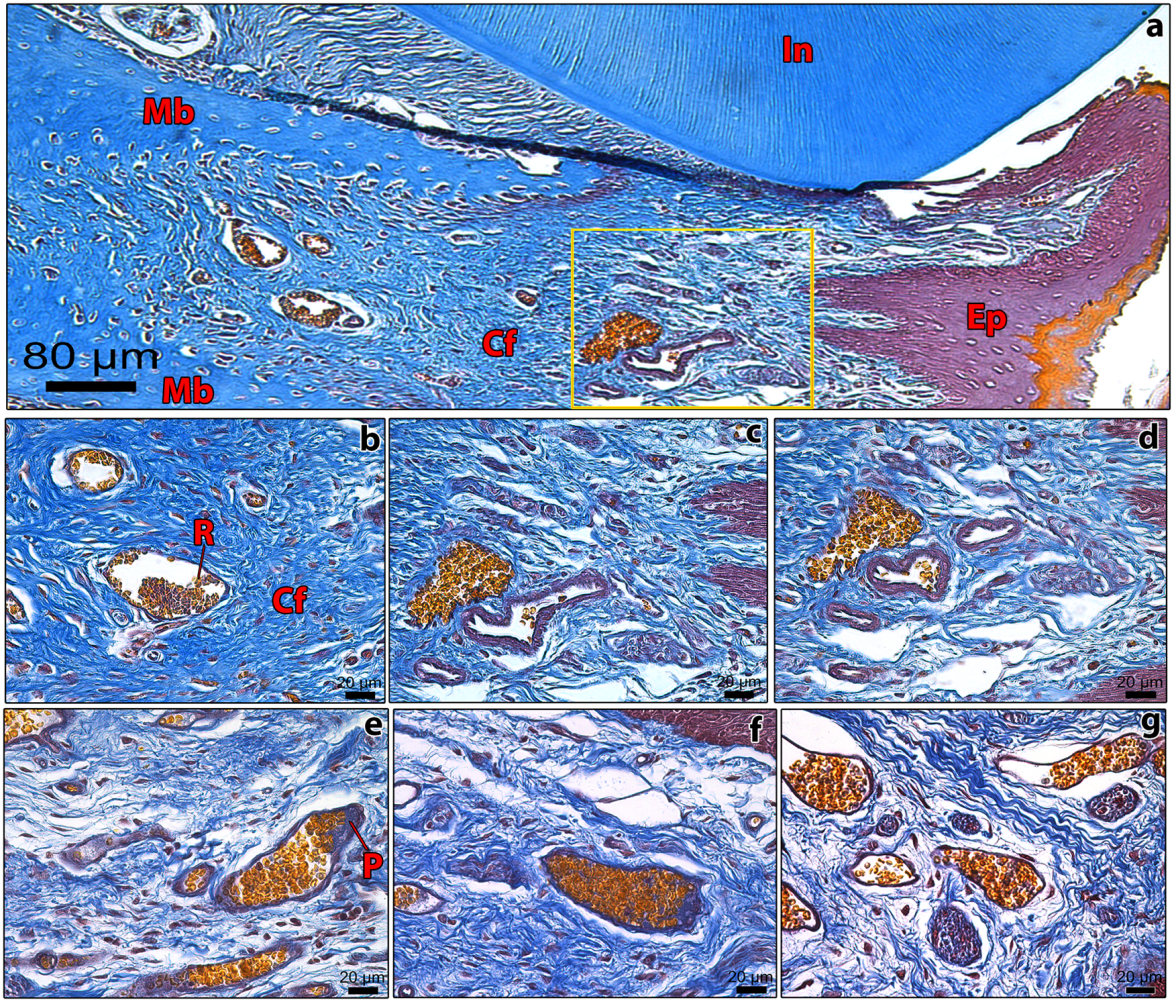
Photomicrographs of transversal sections from the mentolabial region of hamsters, from experimental (boxes a-d) and control (box e-g) groups depicting the gingiva between central incisors (In). The needle was inserted in the rostral region of the interdental gingiva of experimental hamsters; control guinea pigs were not phlebotomized. Sections were stained by Carstairs’ method: collagen fibers (**Cf**) are stained in celeste blue, epithelium (**Ep**) in magenta/purple, red blood cells (**R**), keratin in yellow, and blood platelets (**P**) in light violet. It is also observed the mandible bone (**Mb**) in box **a**. The area around the gingival vein, delineated by the yellow frame in box **a**, is magnified in box **c**. In boxes **b-d**, notice the normal distribution and quantity of collagen fibers, and the absence of inflammatory cells or exudate in lamina propria. Red blood cells were evidenced inside blood vessel lumina in both control (boxes **e**–**g**) and experimental animals (boxes **a**–**d**). Box **a**, bar size = 80 μm; boxes **b**–**g**, bar size = 20 μm.

Histologically, morphological structures in the interdental gingiva of hamsters were very similar to those of guinea pigs (Fig 6). The lamina propria had an intense blood supply, as evidenced by large numbers of small blood vessels. In the more rostral region of the lamina propria, close to the inferior lip, where the needle was initially inserted, there was a typical pattern of venous congestion, even in control animals (Fig 6). Both groups had well preserved epithelium, lamina propria and blood vessels, and no cellular alterations, so that no important morphological differences between experimental and control groups could be demonstrated. Therefore, histological results from guinea pigs and hamsters evidenced that the procedure of multiple venipuncture did not retard wound healing and was minimally aggressive to animals.

Although we deliberately decided to study a small number of animals, which could be considered a limitation of this study, herein we clearly demonstrate that multiple blood sampling from the gingival vein causes minimal, if any, adverse effects to animal welfare. We also observed that the venous access in guinea pigs is more troublesome than in hamsters, but training can overcome this difficulty.

In conclusion, our findings show that weekly blood collection over six weeks can be safely and humanely taken from the gingival vein from both guinea pigs and hamsters. Recently, a similar procedure has been used for blood collection in hamsters experimentally infected with *Leptospira* sp. [53], and it was also showed to be inoffensive for them. Thus, gingival vein puncture is minimally invasive, allows easy venous access and causes few local and systemic injuries. Moreover, to associate it with general anesthetic procedures and other ancillary humane treatment, it is a mean to refine the procedure of blood sampling in hamsters and guinea pigs. For animal procedures requiring multiple blood sampling, such as those for basic sciences or vaccine testing, this venous access route may reduce the number of used animals and decrease their distress. This new venous access route will particularly benefit scientific studies that evaluate the raising of antibodies after immunization, such as those that require the determination of the immunogenic potency of human vaccines.
